# Supplementary material for: Biodiversity stabilizes plant communities through statistical-averaging effects rather than compensatory dynamics
Source: Nat Commun. 2022 Dec 17;13:7804. doi: 10.1038/s41467-022-35514-9 (PMC9759569; doi:10.1038/s41467-022-35514-9)
Supplement: Supplementary file 1 — Supplementary Information [file 41467_2022_35514_MOESM1_ESM.pdf]

# Supplementary Information

# Biodiversity stabilizes plant communities through statistical-averaging effects rather than compensatory dynamics


Lei Zhao[1,*], Shaopeng Wang[2,*], Ruohong Shen[1], Ying Gong[1], Chong Wang[1,*], Pubin Hong[2], and Daniel C. Reuman[3]

[1] *Beijing Key Laboratory of Biodiversity and Organic Farming, College of Resources and Environmental Sciences, China Agricultural University, Beijing 100193 China*

[2] *Institute of Ecology, Key Laboratory for Earth Surface Processes of the Ministry of Education, College of Urban and Environmental Sciences, Peking University, Beijing 100871 China*

[3] *Department of Ecology and Evolutionary Biology and Center for Ecological Research, University of Kansas, Higuchi Hall, 2101 Constant Avenue, Lawrence, Kansas 66047 USA*

*Corresponding authors: Lei Zhao (lei.zhao@cau.edu.cn), Shaopeng Wang (shaopeng.wang@pku.edu.cn), and Chong Wang (wangchong@cau.edu.cn)

**Supplementary Table 1: Significance of compensatory effects (*CPE*).** Within each plot, the significance of *CPE* was assessed as compared to asynchronous surrogates (Methods). Each plot was deemed to have significantly compensatory dynamics (*CPE* significantly greater than 1), or significantly synchronous dynamics (*CPE* significantly less than 1), or neither.

| Dataset | Site | Plots | Proportion of plots showing significantly compensatory dynamics | Nonsignificance | Proportion of plots showing significantly synchronous dynamics |
|---|---|---|---|---|---|
| Plant Survey Dataset | JRG | 18 | 100% | 0% | 0% |
| | KBS | 30 | 30% | 70% | 0% |
| | HAY | 13 | 46.2% | 53.8% | 0% |
| | JRN | 47 | 12.5% | 83.3% | 4.2% |
| | KNZ | 20 | 5% | 90% | 5% |
| | SEV | 22 | 0% | 90.9% | 9.1% |
| | CDR | 5 | 0% | 100% | 0% |
| | SGS | 100 | 14% | 86% | 0% |
| | VC | 6 | 66.7% | 33.3% | 0% |
| Biodiversity-manipulated Experiment Dataset | BigBio | 122 | 4.8% | 86.4% | 8.8% |
| | BioCON | 42 | 30.5% | 69.5% | 0% |

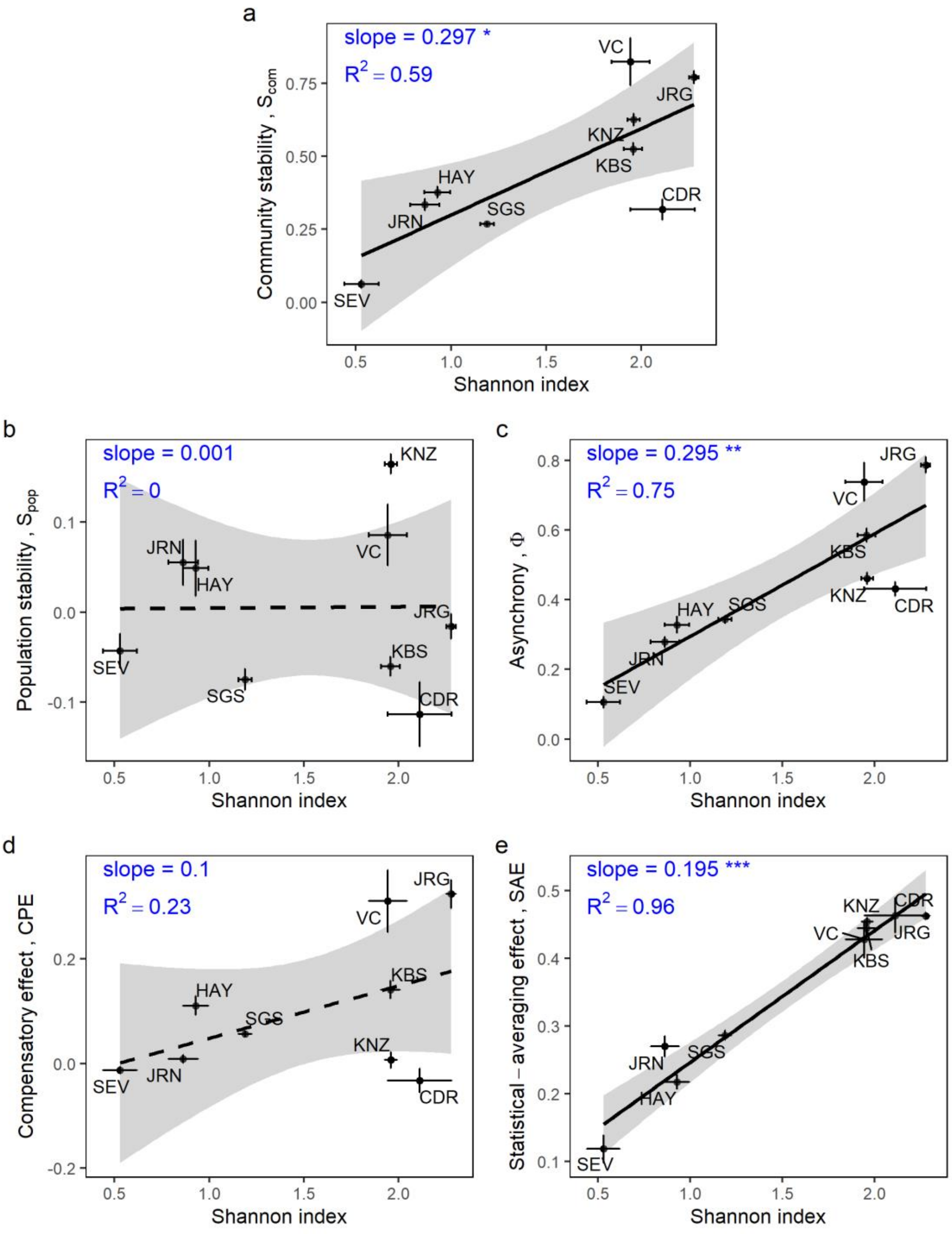


**Supplementary Fig. 1: Effects of the Shannon index on community stability and its components across the nine sites in the plant survey dataset.** Relationships are shown between the Shannon index and (**a**) community stability ($p$ = 0.015), (**b**) population stability ($p$ = 0.979), (**c**) asynchrony ($p$ = 0.003), (**d**) the compensatory effect ($p$ = 0.189), and (**e**) the statistical-averaging effect ($p$ < 0.001). All values (including standard error) are $\log_{10}$ transformed except for the Shannon index. Regression and significance results formatted as in Fig. 2.

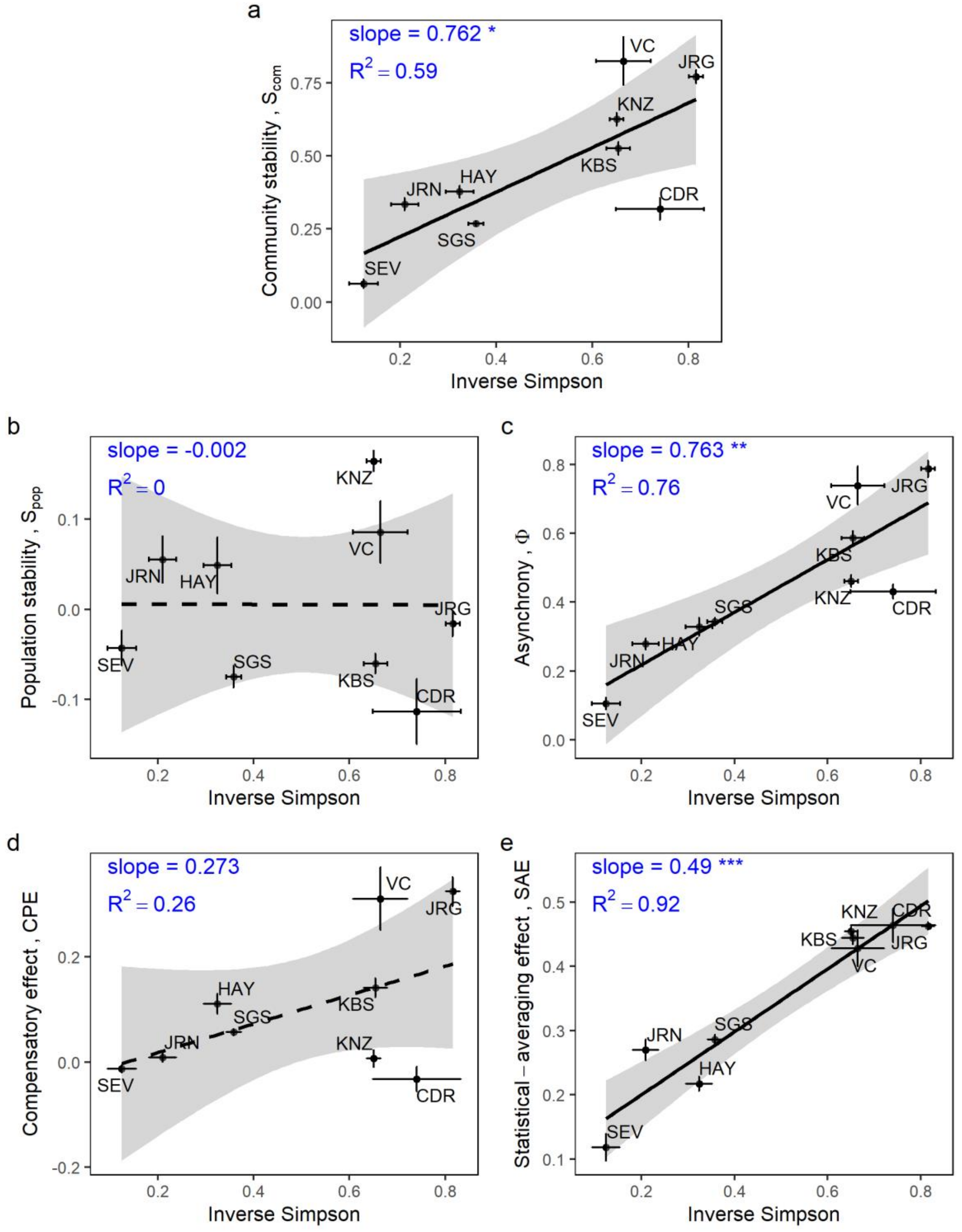


**Supplementary Fig. 2: Effects of the inverse of Simpson's index on community stability and its components across the nine sites in the plant survey dataset.** Relationships are shown between the inverse of Simpson's index and (a) community stability ($p = 0.016$), (b) population stability ($p = 0.991$), (c) asynchrony ($p = 0.002$), (d) the compensatory effect ($p = 0.160$), and (e) the statistical-averaging effect ($p < 0.0001$). All values (including standard error) are $\log_{10}$ transformed. Regression and significance results formatted as in Fig. 2.

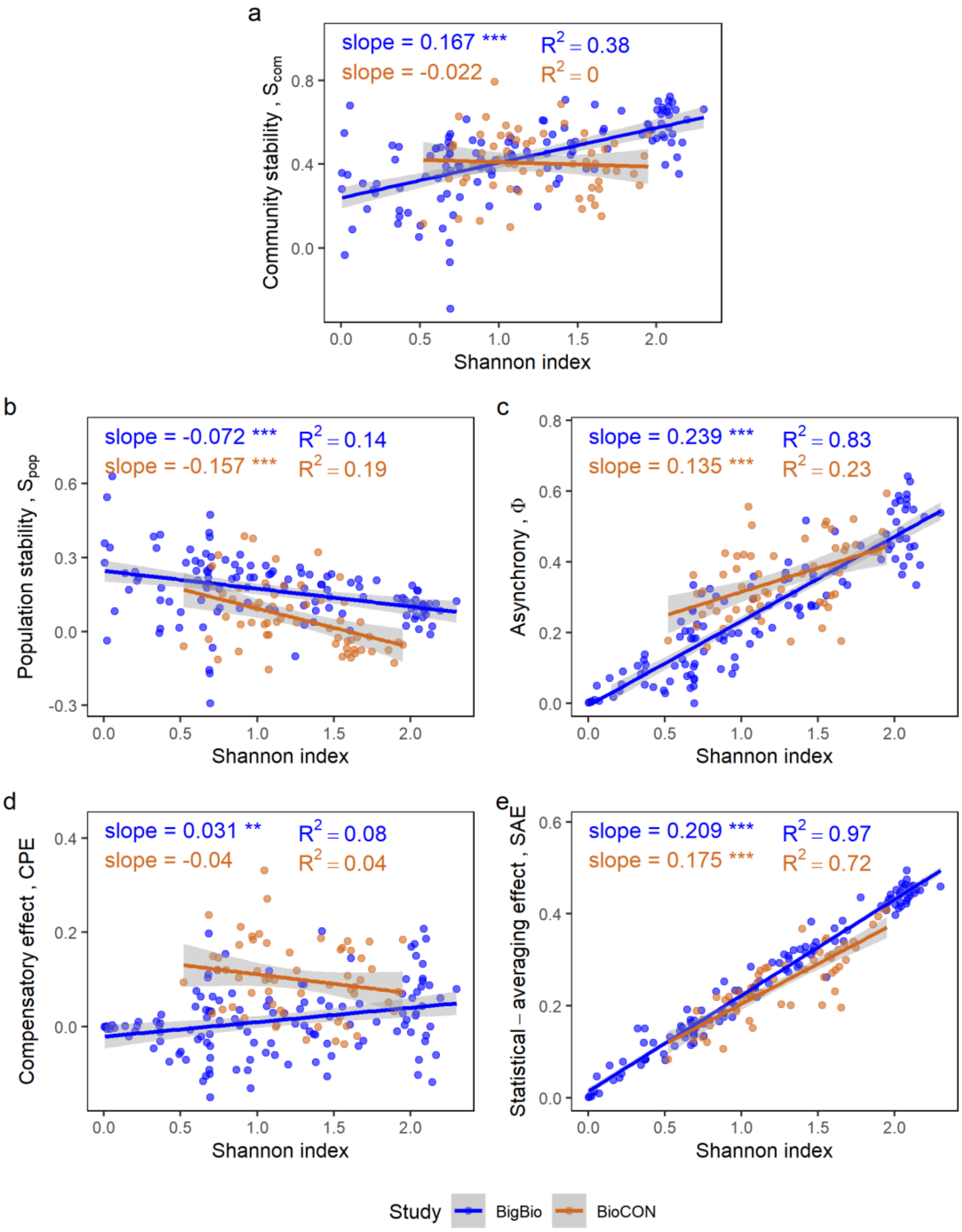


**Supplementary Fig. 3: Effects of the Shannon index on community stability and its components in the biodiversity-manipulated experimental dataset.** Relationships are shown between the Shannon index and (**a**) community stability ($p < 0.001$ for BigBio and $p = 0.675$ for BioCON), (**b**) population stability ($p < 0.001$ for both studies), (**c**) asynchrony ($p < 0.001$ for both studies), (**d**) the compensatory effect ($p = 0.002$ for BigBio and $p = 0.147$ for BioCON), and (**e**) the statistical-averaging effect ($p < 0.001$ for both studies) within two studies (indicated by different colours). All values are $\log_{10}$ transformed except for the Shannon index. Regression and significance results formatted as in Fig. 2.

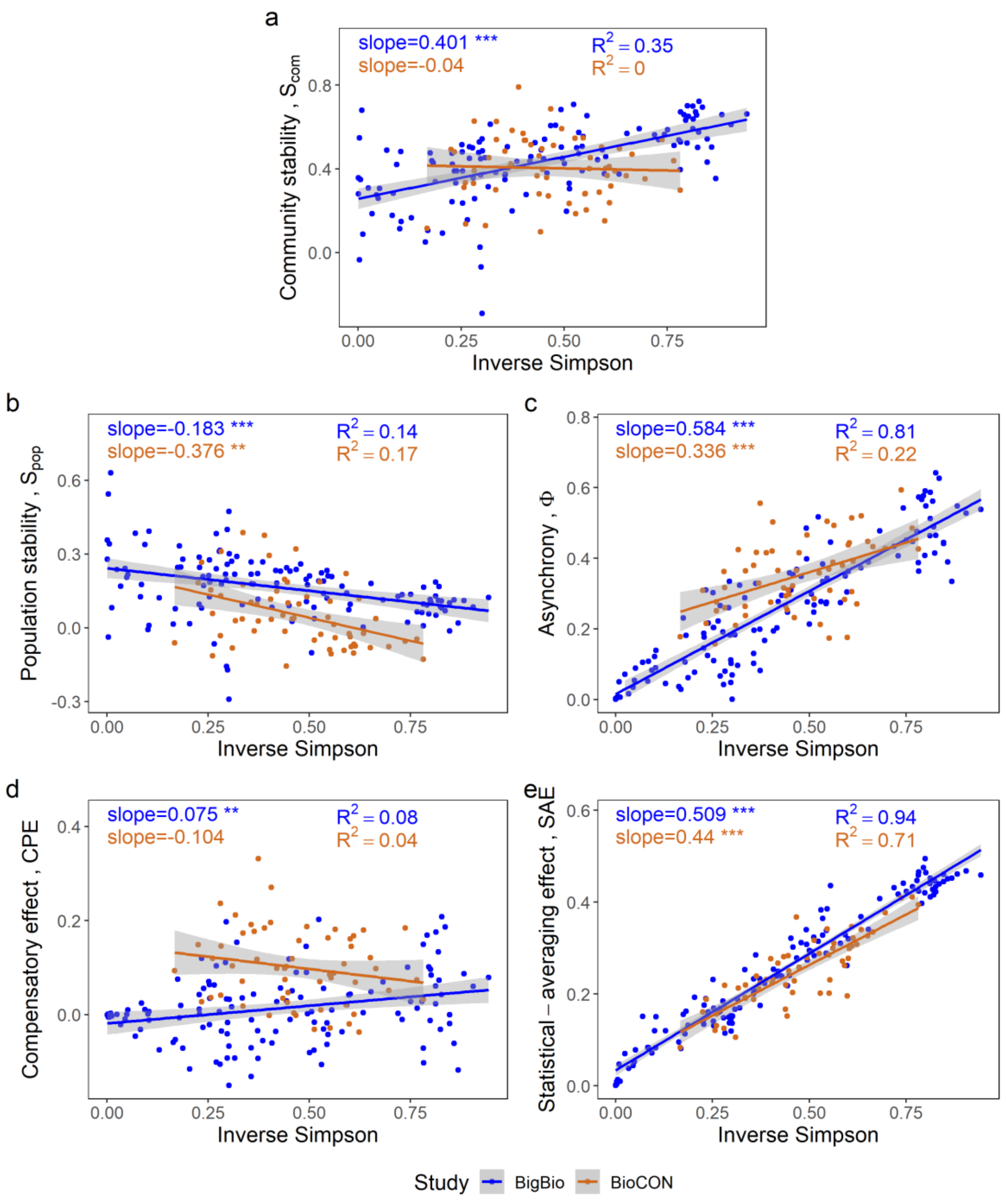


**Supplementary Fig. 4: Effects of inverse Simpson's index on community stability and its components in the biodiversity-manipulated experimental dataset.** Relationships are shown between the inverse Simpson's index and (**a**) community stability ($p < 0.001$ for BigBio and $p = 0.769$ for BioCON), (**b**) population stability ($p < 0.001$ for BigBio and $p = 0.001$ for BioCON), (**c**) asynchrony ($p < 0.001$ for both studies), (**d**) the compensatory effect ($p = 0.002$ for BigBio and $p = 0.147$ for BioCON), and (**e**) the statistical-averaging effect ($p < 0.001$ for both studies) within two studies (indicated by different colours). All values are log10 transformed. Regression and significance results formatted as in Fig. 2.

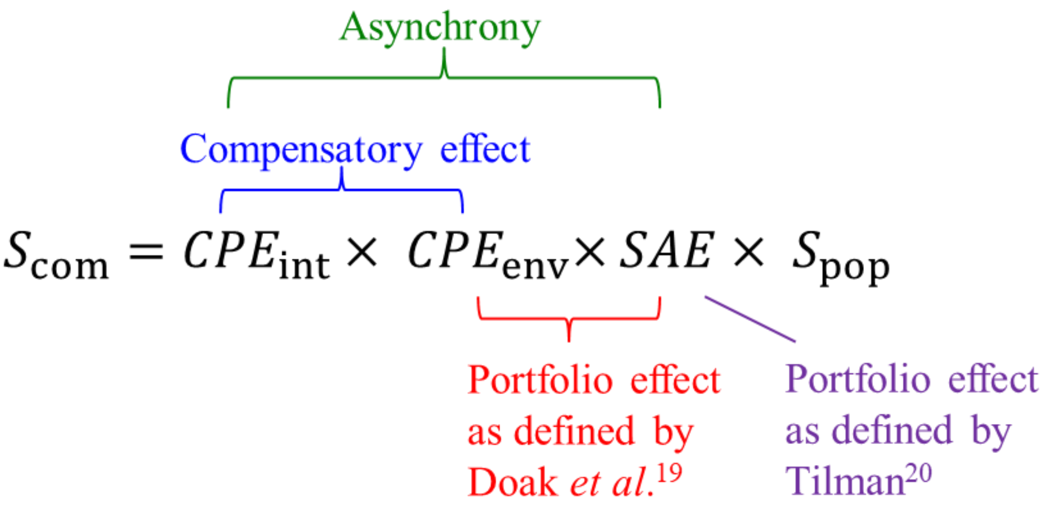


**Supplementary Fig. 5: Summary equation of our full decomposition of community stability, and ecological meanings of components.**

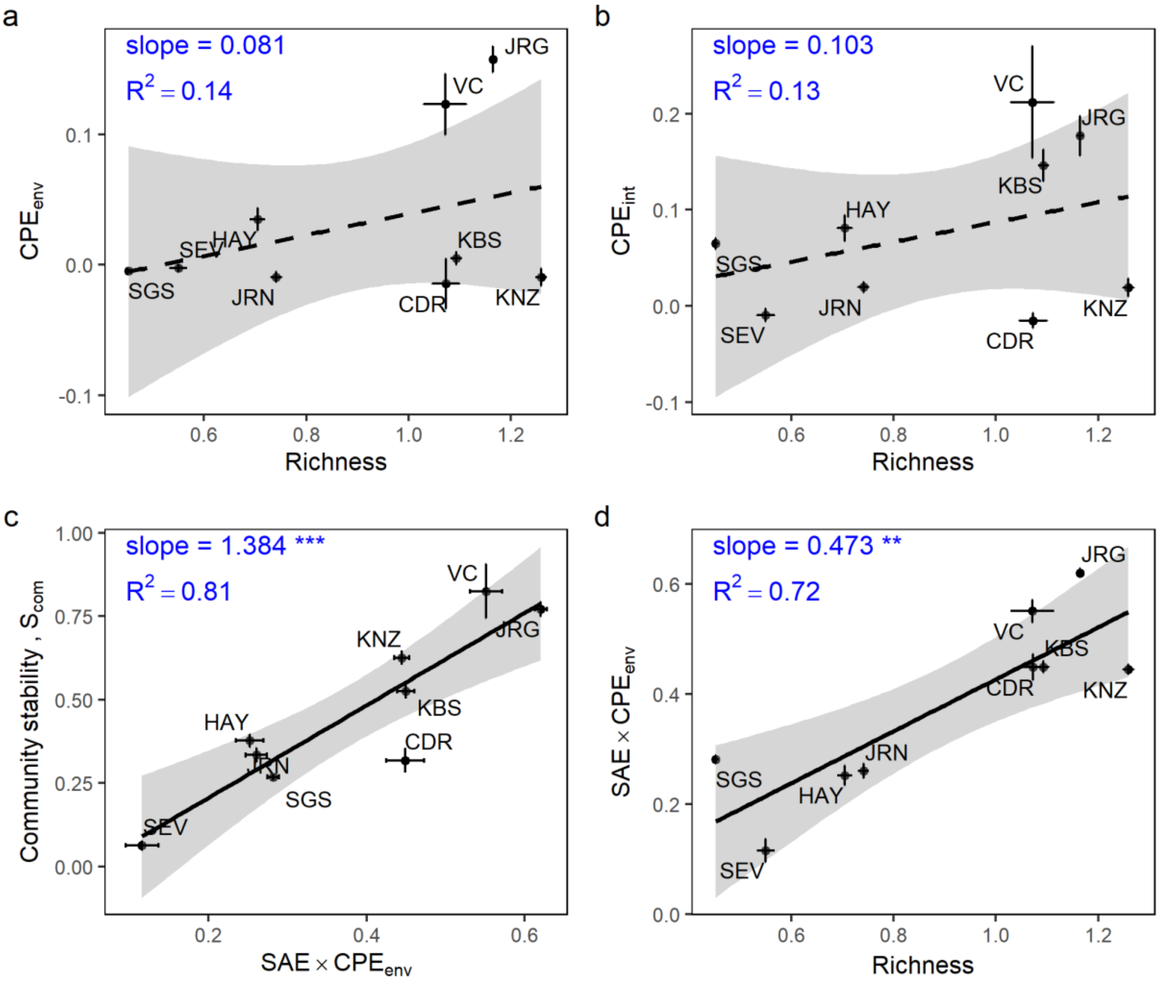


**Supplementary Fig. 6: Relationships involving compensatory effects due to joint responses to environment fluctuations ($CPE_{env}$) and due to species interactions ($CPE_{int}$) across the nine sites in the plant survey dataset.** Relationships are shown between (**a**) $CPE_{env}$ and richness ($p$ = 0.329), (**b**) $CPE_{int}$ and richness ($p$ = 0.342), (**c**) community stability and the product of statistical averaging effect and $CPE_{env}$ ($p$ < 0.001), and (**d**) richness and the product of statistical averaging effect and $CPE_{env}$ ($p$ = 0.004). All values (including standard errors) are $\log_{10}$ transformed. Regression and significance results formatted as in Fig. 2.

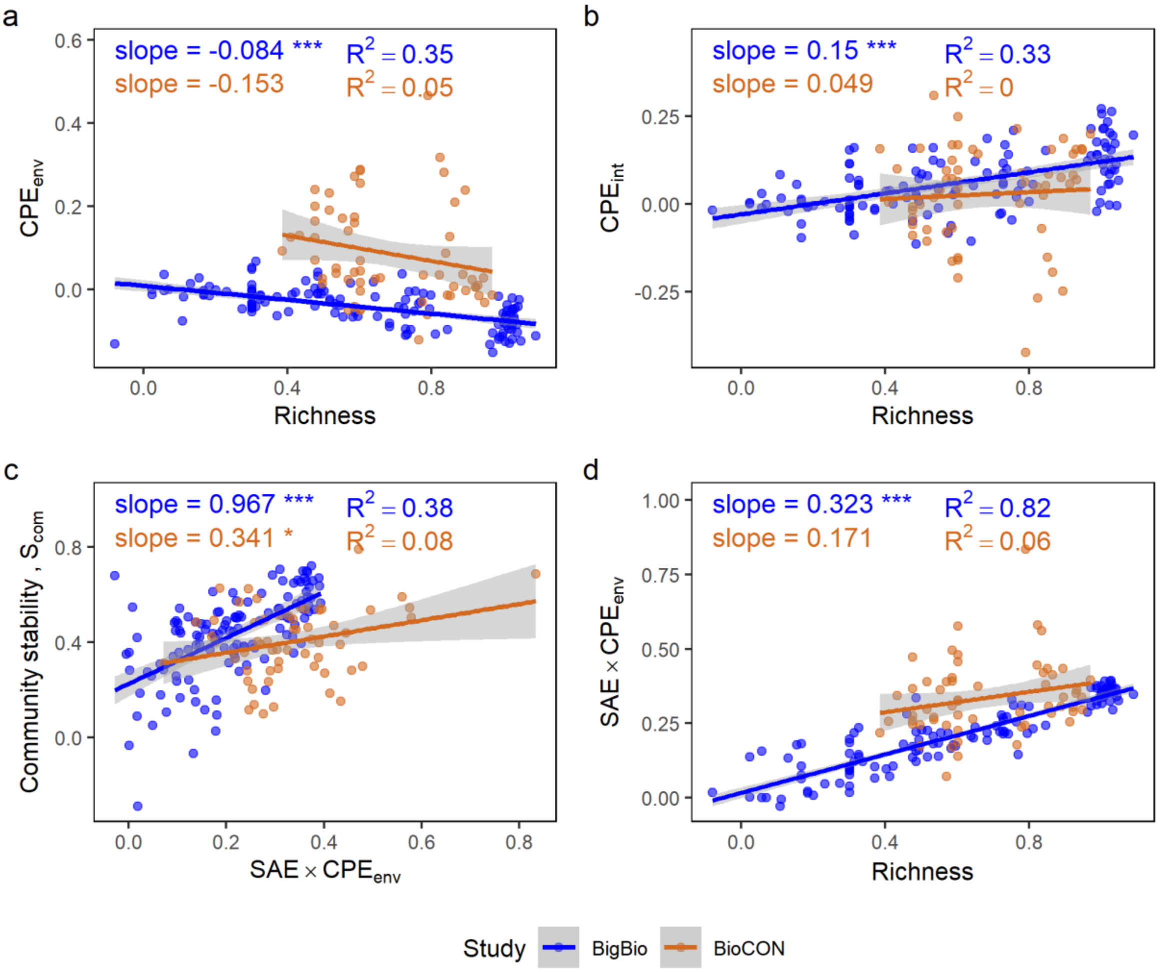


**Supplementary Fig. 7: Relationships involving compensatory effects due to joint responses to environment fluctuations (*CPE*$_{\text{env}}$) and due to species interactions (*CPE*$_{\text{int}}$) within studies of the biodiversity-manipulated experimental dataset.** Relationships are shown between (**a**) *CPE*$_{\text{env}}$ and richness ($p < 0.001$ for BigBio and $p = 0.094$ for BioCON), (**b**) *CPE*$_{\text{int}}$ and richness ($p < 0.001$ for BigBio and $p = 0.653$ for BioCON), (**c**) community stability and the product of statistical averaging effect and *CPE*$_{\text{env}}$ ($p < 0.001$ for BigBio and $p = 0.029$ for BioCON), and (**d**) richness and the product of statistical averaging effect and *CPE*$_{\text{env}}$ (($p < 0.001$ for BigBio and $p = 0.072$ for BioCON)). All values (including standard errors) are $\log_{10}$ transformed. Regression and significance results formatted as in Fig. 2.
